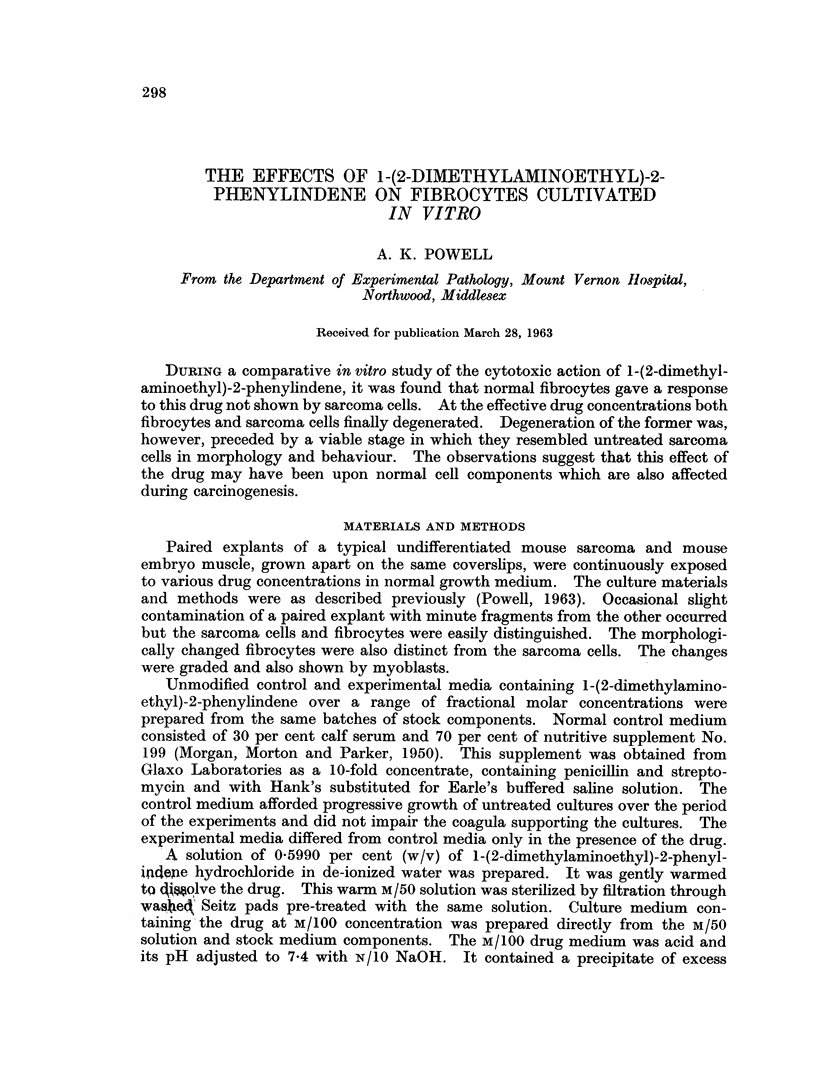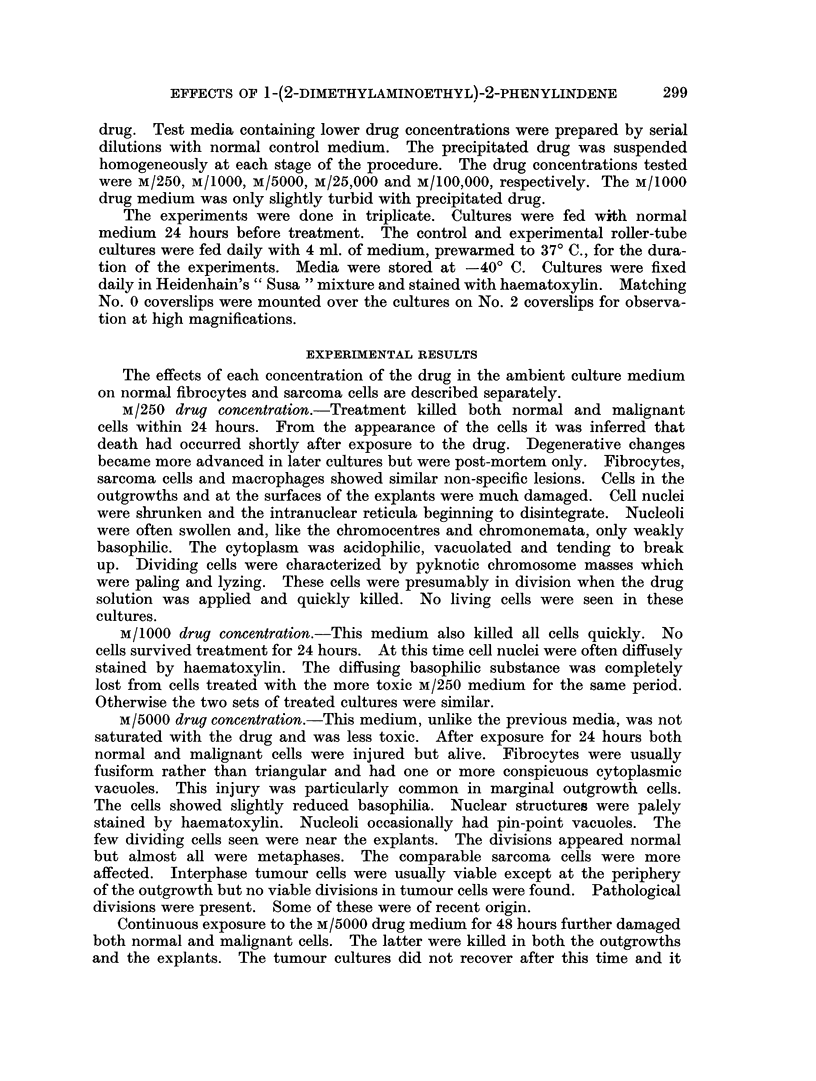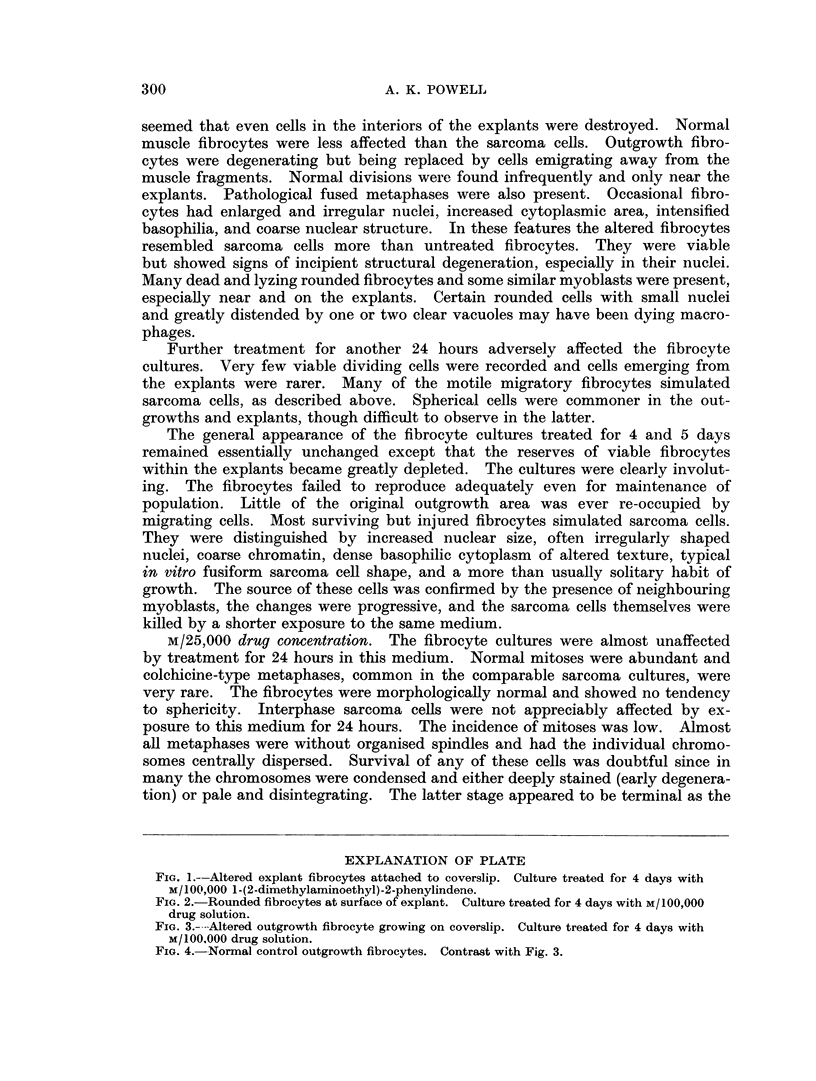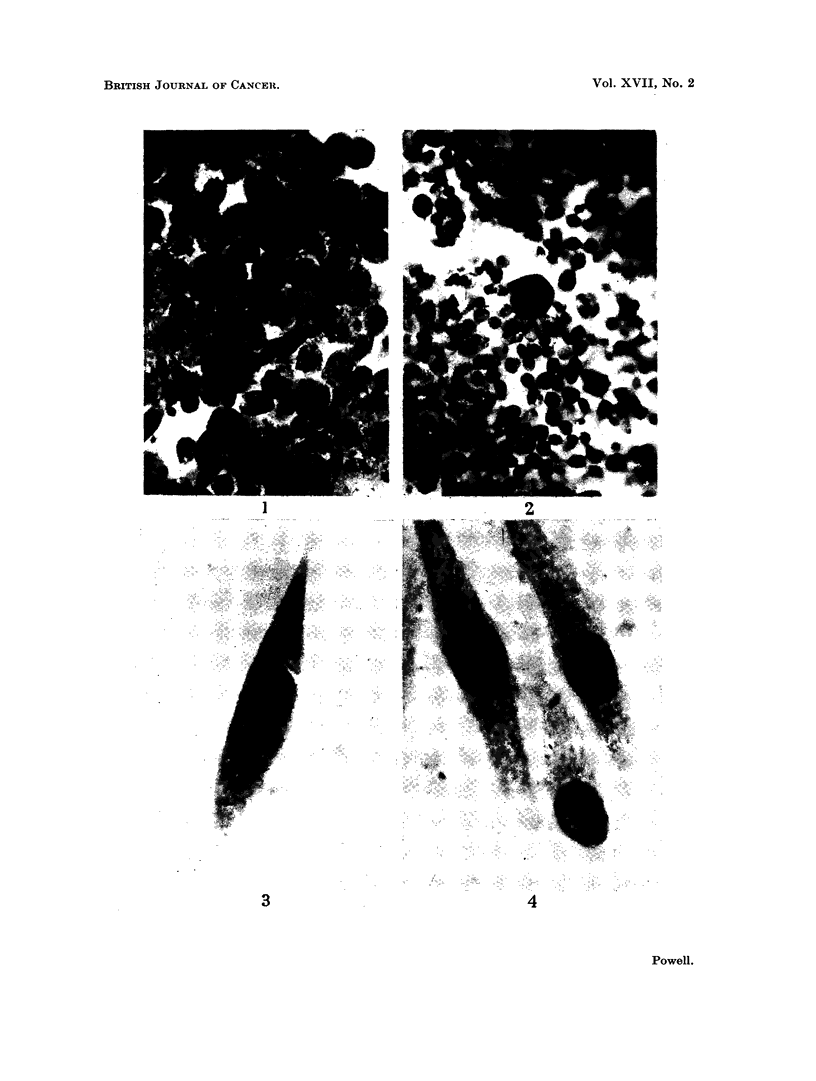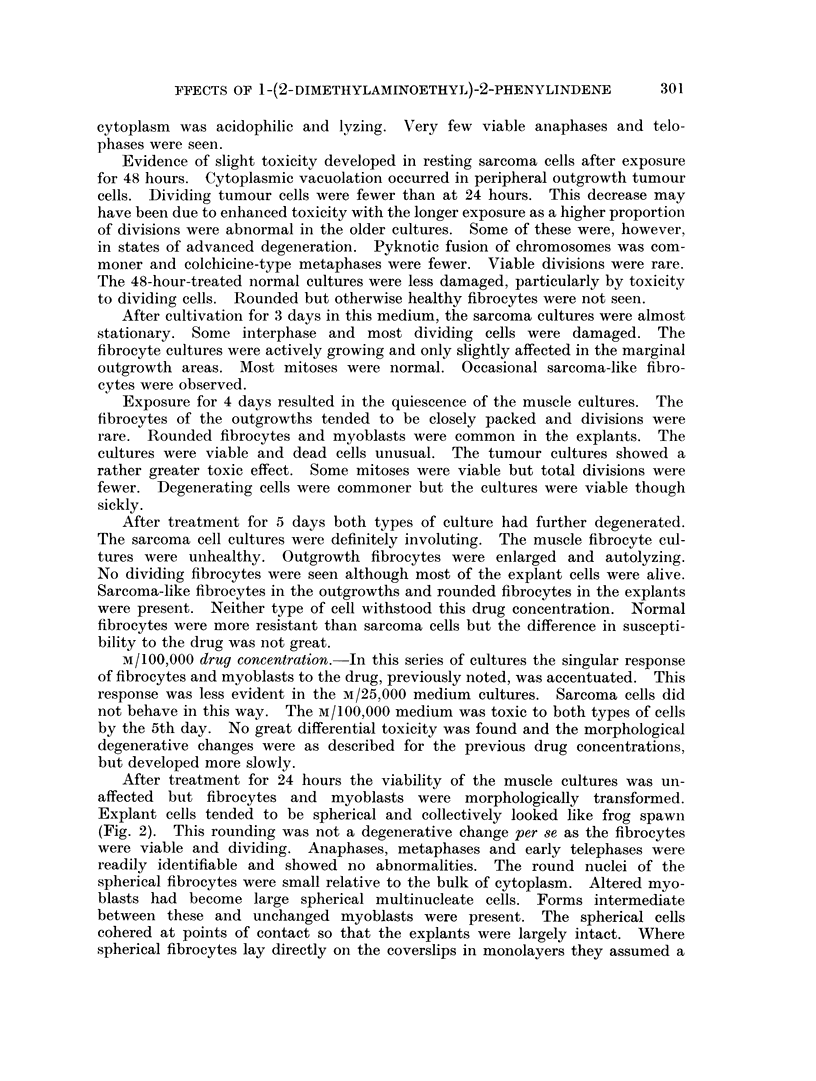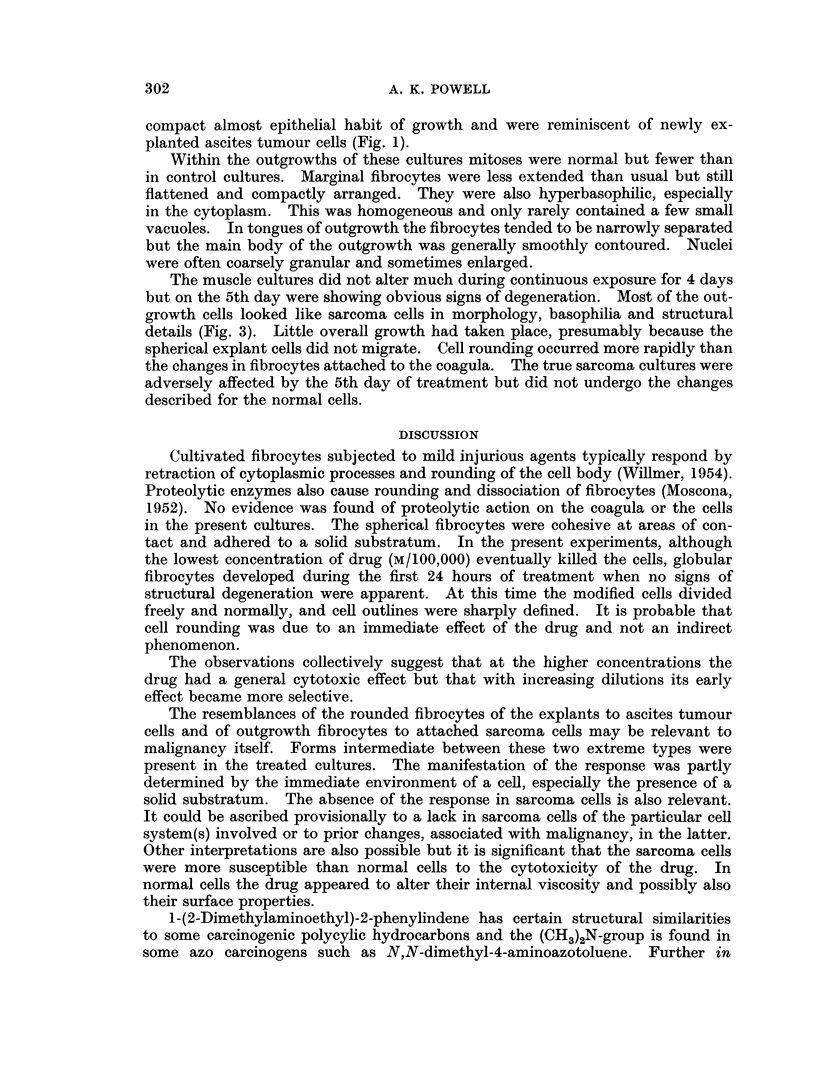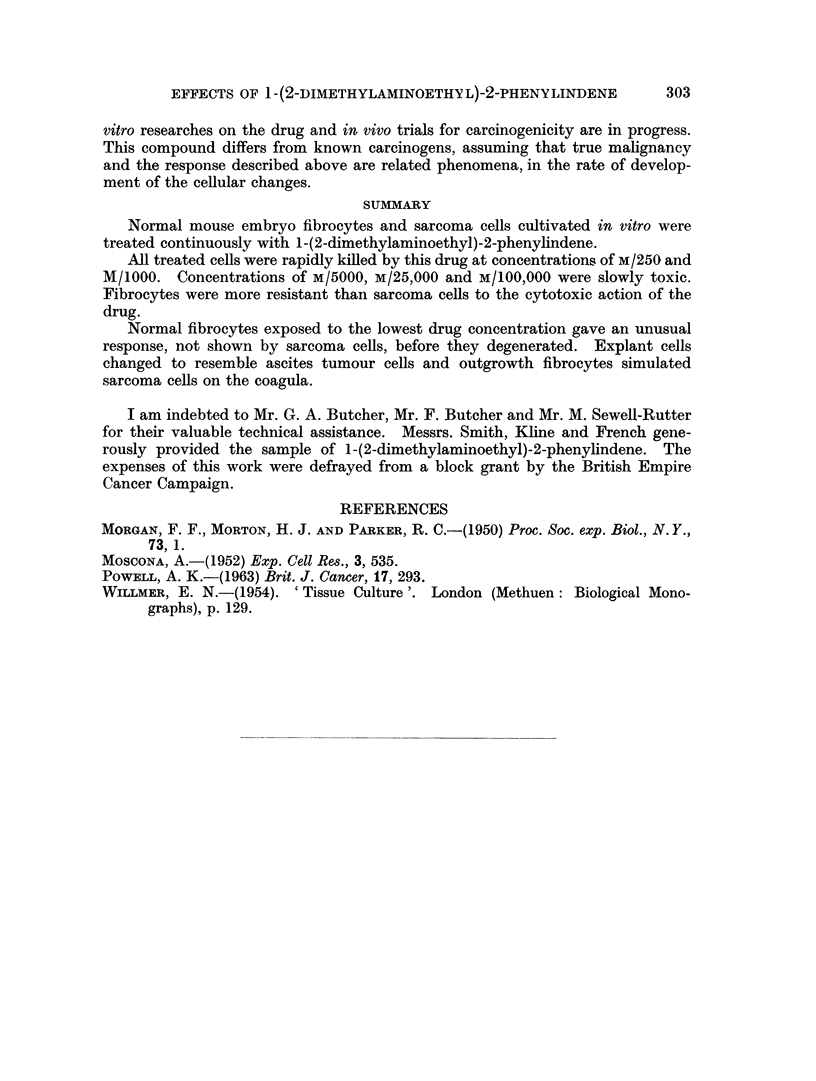# The Effects of 1-(2-Dimethylaminoethyl)-2-Phenylindene on Fibrocytes Cultivated In Vitro

**DOI:** 10.1038/bjc.1963.43

**Published:** 1963-06

**Authors:** A. K. Powell

## Abstract

**Images:**


### THE EFFECTS OF 1-(2-DIMETHYLAMINOETHYL)-2-PHENYLINDENE ON FIBROCYTES CULTIVATED *IN VITRO*

A. K. POWELL

*From the Department of Experimental Pathology, Mount Vernon Hospital, Northwood, Middlesex*

Received for publication March 28, 1963

DURING a comparative *in vitro* study of the cytotoxic action of 1-(2-dimethylaminoethyl)-2-phenylindene, it was found that normal fibrocytes gave a response to this drug not shown by sarcoma cells. At the effective drug concentrations both fibrocytes and sarcoma cells finally degenerated. Degeneration of the former was, however, preceded by a viable stage in which they resembled untreated sarcoma cells in morphology and behaviour. The observations suggest that this effect of the drug may have been upon normal cell components which are also affected during carcinogenesis.

### MATERIALS AND METHODS

Paired explants of a typical undifferentiated mouse sarcoma and mouse embryo muscle, grown apart on the same coverslips, were continuously exposed to various drug concentrations in normal growth medium. The culture materials and methods were as described previously (Powell, 1963). Occasional slight contamination of a paired explant with minute fragments from the other occurred but the sarcoma cells and fibrocytes were easily distinguished. The morphologically changed fibrocytes were also distinct from the sarcoma cells. The changes were graded and also shown by myoblasts.

Unmodified control and experimental media containing 1-(2-dimethylaminoethyl)-2-phenylindene over a range of fractional molar concentrations were prepared from the same batches of stock components. Normal control medium consisted of 30 per cent calf serum and 70 per cent of nutritive supplement No. 199 (Morgan, Morton and Parker, 1950). This supplement was obtained from Glaxo Laboratories as a 10-fold concentrate, containing penicillin and streptomycin and with Hank's substituted for Earle's buffered saline solution. The control medium afforded progressive growth of untreated cultures over the period of the experiments and did not impair the coagula supporting the cultures. The experimental media differed from control media only in the presence of the drug.

A solution of 0·5990 per cent (w/v) of 1-(2-dimethylaminoethyl)-2-phenylindene hydrochloride in de-ionized water was prepared. It was gently warmed to dissolve the drug. This warm M/50 solution was sterilized by filtration through washed Seitz pads pre-treated with the same solution. Culture medium containing the drug at M/100 concentration was prepared directly from the M/50 solution and stock medium components. The M/100 drug medium was acid and its pH adjusted to 7·4 with N/10 NaOH. It contained a precipitate of excess drug. Test media containing lower drug concentrations were prepared by serial dilutions with normal control medium. The precipitated drug was suspended homogeneously at each stage of the procedure. The drug concentrations tested were M/250, M/1000, M/5000, M/25,000 and M/100,000, respectively. The M/1000 drug medium was only slightly turbid with precipitated drug.

The experiments were done in triplicate. Cultures were fed with normal medium 24 hours before treatment. The control and experimental roller-tube cultures were fed daily with 4 ml. of medium, prewarmed to 37° C., for the duration of the experiments. Media were stored at —40° C. Cultures were fixed daily in Heidenhain's " Susa " mixture and stained with haematoxylin. Matching No. 0 coverslips were mounted over the cultures on No. 2 coverslips for observation at high magnifications.

### EXPERIMENTAL RESULTS

The effects of each concentration of the drug in the ambient culture medium on normal fibrocytes and sarcoma cells are described separately.

M/250 *drug concentration.*—Treatment killed both normal and malignant cells within 24 hours. From the appearance of the cells it was inferred that death had occurred shortly after exposure to the drug. Degenerative changes became more advanced in later cultures but were post-mortem only. Fibrocytes, sarcoma cells and macrophages showed similar non-specific lesions. Cells in the outgrowths and at the surfaces of the explants were much damaged. Cell nuclei were shrunken and the intranuclear reticula beginning to disintegrate. Nucleoli were often swollen and, like the chromocentres and chromonemata, only weakly basophilic. The cytoplasm was acidophilic, vacuolated and tending to break up. Dividing cells were characterized by pyknotic chromosome masses which were paling and lyzing. These cells were presumably in division when the drug solution was applied and quickly killed. No living cells were seen in these cultures.

M/1000 *drug concentration.*—This medium also killed all cells quickly. No cells survived treatment for 24 hours. At this time cell nuclei were often diffusely stained by haematoxylin. The diffusing basophilic substance was completely lost from cells treated with the more toxic M/250 medium for the same period. Otherwise the two sets of treated cultures were similar.

M/5000 *drug concentration.*—This medium, unlike the previous media, was not saturated with the drug and was less toxic. After exposure for 24 hours both normal and malignant cells were injured but alive. Fibrocytes were usually fusiform rather than triangular and had one or more conspicuous cytoplasmic vacuoles. This injury was particularly common in marginal outgrowth cells. The cells showed slightly reduced basophilia. Nuclear structures were palely stained by haematoxylin. Nucleoli occasionally had pin-point vacuoles. The few dividing cells seen were near the explants. The divisions appeared normal but almost all were metaphases. The comparable sarcoma cells were more affected. Interphase tumour cells were usually viable except at the periphery of the outgrowth but no viable divisions in tumour cells were found. Pathological divisions were present. Some of these were of recent origin.

Continuous exposure to the M/5000 drug medium for 48 hours further damaged both normal and malignant cells. The latter were killed in both the outgrowths and the explants. The tumour cultures did not recover after this time and it seemed that even cells in the interiors of the explants were destroyed. Normal muscle fibrocytes were less affected than the sarcoma cells. Outgrowth fibrocytes were degenerating but being replaced by cells emigrating away from the muscle fragments. Normal divisions were found infrequently and only near the explants. Pathological fused metaphases were also present. Occasional fibrocytes had enlarged and irregular nuclei, increased cytoplasmic area, intensified basophilia, and coarse nuclear structure. In these features the altered fibrocytes resembled sarcoma cells more than untreated fibrocytes. They were viable but showed signs of incipient structural degeneration, especially in their nuclei. Many dead and lyzing rounded fibrocytes and some similar myoblasts were present, especially near and on the explants. Certain rounded cells with small nuclei and greatly distended by one or two clear vacuoles may have been dying macrophages.

Further treatment for another 24 hours adversely affected the fibrocyte cultures. Very few viable dividing cells were recorded and cells emerging from the explants were rarer. Many of the motile migratory fibrocytes simulated sarcoma cells, as described above. Spherical cells were commoner in the outgrowths and explants, though difficult to observe in the latter.

The general appearance of the fibrocyte cultures treated for 4 and 5 days remained essentially unchanged except that the reserves of viable fibrocytes within the explants became greatly depleted. The cultures were clearly involuting. The fibrocytes failed to reproduce adequately even for maintenance of population. Little of the original outgrowth area was ever re-occupied by migrating cells. Most surviving but injured fibrocytes simulated sarcoma cells. They were distinguished by increased nuclear size, often irregularly shaped nuclei, coarse chromatin, dense basophilic cytoplasm of altered texture, typical *in vitro* fusiform sarcoma cell shape, and a more than usually solitary habit of growth. The source of these cells was confirmed by the presence of neighbouring myoblasts, the changes were progressive, and the sarcoma cells themselves were killed by a shorter exposure to the same medium.

M/25,000 *drug concentration.* The fibrocyte cultures were almost unaffected by treatment for 24 hours in this medium. Normal mitoses were abundant and colchicine-type metaphases, common in the comparable sarcoma cultures, were very rare. The fibrocytes were morphologically normal and showed no tendency to sphericity. Interphase sarcoma cells were not appreciably affected by exposure to this medium for 24 hours. The incidence of mitoses was low. Almost all metaphases were without organised spindles and had the individual chromosomes centrally dispersed. Survival of any of these cells was doubtful since in many the chromosomes were condensed and either deeply stained (early degeneration) or pale and disintegrating. The latter stage appeared to be terminal as the

---

EXPLANATION OF PLATE

FIG. 1.—Altered explant fibrocytes attached to coverslip. Culture treated for 4 days with M/100,000 1-(2-dimethylaminoethyl)-2-phenylindene.

FIG. 2.—Rounded fibrocytes at surface of explant. Culture treated for 4 days with M/100,000 drug solution.

FIG. 3.—Altered outgrowth fibrocyte growing on coverslip. Culture treated for 4 days with M/100,000 drug solution.

FIG. 4.—Normal control outgrowth fibrocytes. Contrast with Fig. 3.

1 2 3 4

Powell.

cytoplasm was acidophilic and lyzing. Very few viable anaphases and telophases were seen.

Evidence of slight toxicity developed in resting sarcoma cells after exposure for 48 hours. Cytoplasmic vacuolation occurred in peripheral outgrowth tumour cells. Dividing tumour cells were fewer than at 24 hours. This decrease may have been due to enhanced toxicity with the longer exposure as a higher proportion of divisions were abnormal in the older cultures. Some of these were, however, in states of advanced degeneration. Pyknotic fusion of chromosomes was commoner and colchicine-type metaphases were fewer. Viable divisions were rare. The 48-hour-treated normal cultures were less damaged, particularly by toxicity to dividing cells. Rounded but otherwise healthy fibrocytes were not seen.

After cultivation for 3 days in this medium, the sarcoma cultures were almost stationary. Some interphase and most dividing cells were damaged. The fibrocyte cultures were actively growing and only slightly affected in the marginal outgrowth areas. Most mitoses were normal. Occasional sarcoma-like fibrocytes were observed.

Exposure for 4 days resulted in the quiescence of the muscle cultures. The fibrocytes of the outgrowths tended to be closely packed and divisions were rare. Rounded fibrocytes and myoblasts were common in the explants. The cultures were viable and dead cells unusual. The tumour cultures showed a rather greater toxic effect. Some mitoses were viable but total divisions were fewer. Degenerating cells were commoner but the cultures were viable though sickly.

After treatment for 5 days both types of culture had further degenerated. The sarcoma cell cultures were definitely involuting. The muscle fibrocyte cultures were unhealthy. Outgrowth fibrocytes were enlarged and autolyzing. No dividing fibrocytes were seen although most of the explant cells were alive. Sarcoma-like fibrocytes in the outgrowths and rounded fibrocytes in the explants were present. Neither type of cell withstood this drug concentration. Normal fibrocytes were more resistant than sarcoma cells but the difference in susceptibility to the drug was not great.

M/100,000 *drug concentration.*—In this series of cultures the singular response of fibrocytes and myoblasts to the drug, previously noted, was accentuated. This response was less evident in the M/25,000 medium cultures. Sarcoma cells did not behave in this way. The M/100,000 medium was toxic to both types of cells by the 5th day. No great differential toxicity was found and the morphological degenerative changes were as described for the previous drug concentrations, but developed more slowly.

After treatment for 24 hours the viability of the muscle cultures was unaffected but fibrocytes and myoblasts were morphologically transformed. Explant cells tended to be spherical and collectively looked like frog spawn (Fig. 2). This rounding was not a degenerative change *per se* as the fibrocytes were viable and dividing. Anaphases, metaphases and early telephases were readily identifiable and showed no abnormalities. The round nuclei of the spherical fibrocytes were small relative to the bulk of cytoplasm. Altered myoblasts had become large spherical multinucleate cells. Forms intermediate between these and unchanged myoblasts were present. The spherical cells cohered at points of contact so that the explants were largely intact. Where spherical fibrocytes lay directly on the coverslips in monolayers they assumed a compact almost epithelial habit of growth and were reminiscent of newly explanted ascites tumour cells (Fig. 1).

Within the outgrowths of these cultures mitoses were normal but fewer than in control cultures. Marginal fibrocytes were less extended than usual but still flattened and compactly arranged. They were also hyperbasophilic, especially in the cytoplasm. This was homogeneous and only rarely contained a few small vacuoles. In tongues of outgrowth the fibrocytes tended to be narrowly separated but the main body of the outgrowth was generally smoothly contoured. Nuclei were often coarsely granular and sometimes enlarged.

The muscle cultures did not alter much during continuous exposure for 4 days but on the 5th day were showing obvious signs of degeneration. Most of the outgrowth cells looked like sarcoma cells in morphology, basophilia and structural details (Fig. 3). Little overall growth had taken place, presumably because the spherical explant cells did not migrate. Cell rounding occurred more rapidly than the changes in fibrocytes attached to the coagula. The true sarcoma cultures were adversely affected by the 5th day of treatment but did not undergo the changes described for the normal cells.

### DISCUSSION

Cultivated fibrocytes subjected to mild injurious agents typically respond by retraction of cytoplasmic processes and rounding of the cell body (Willmer, 1954). Proteolytic enzymes also cause rounding and dissociation of fibrocytes (Moscona, 1952). No evidence was found of proteolytic action on the coagula or the cells in the present cultures. The spherical fibrocytes were cohesive at areas of contact and adhered to a solid substratum. In the present experiments, although the lowest concentration of drug (M/100,000) eventually killed the cells, globular fibrocytes developed during the first 24 hours of treatment when no signs of structural degeneration were apparent. At this time the modified cells divided freely and normally, and cell outlines were sharply defined. It is probable that cell rounding was due to an immediate effect of the drug and not an indirect phenomenon.

The observations collectively suggest that at the higher concentrations the drug had a general cytotoxic effect but that with increasing dilutions its early effect became more selective.

The resemblances of the rounded fibrocytes of the explants to ascites tumour cells and of outgrowth fibrocytes to attached sarcoma cells may be relevant to malignancy itself. Forms intermediate between these two extreme types were present in the treated cultures. The manifestation of the response was partly determined by the immediate environment of a cell, especially the presence of a solid substratum. The absence of the response in sarcoma cells is also relevant. It could be ascribed provisionally to a lack in sarcoma cells of the particular cell system(s) involved or to prior changes, associated with malignancy, in the latter. Other interpretations are also possible but it is significant that the sarcoma cells were more susceptible than normal cells to the cytotoxicity of the drug. In normal cells the drug appeared to alter their internal viscosity and possibly also their surface properties.

1-(2-Dimethylaminoethyl)-2-phenylindene has certain structural similarities to some carcinogenic polycylic hydrocarbons and the $(CH_3)_2N$-group is found in some azo carcinogens such as *N*,*N*-dimethyl-4-aminoazotoluene. Further *in*

*vitro* researches on the drug and *in vivo* trials for carcinogenicity are in progress. This compound differs from known carcinogens, assuming that true malignancy and the response described above are related phenomena, in the rate of development of the cellular changes.

### SUMMARY

Normal mouse embryo fibrocytes and sarcoma cells cultivated *in vitro* were treated continuously with 1-(2-dimethylaminoethyl)-2-phenylindene.

All treated cells were rapidly killed by this drug at concentrations of M/250 and M/1000. Concentrations of M/5000, M/25,000 and M/100,000 were slowly toxic. Fibrocytes were more resistant than sarcoma cells to the cytotoxic action of the drug.

Normal fibrocytes exposed to the lowest drug concentration gave an unusual response, not shown by sarcoma cells, before they degenerated. Explant cells changed to resemble ascites tumour cells and outgrowth fibrocytes simulated sarcoma cells on the coagula.

I am indebted to Mr. G. A. Butcher, Mr. F. Butcher and Mr. M. Sewell-Rutter for their valuable technical assistance. Messrs. Smith, Kline and French generously provided the sample of 1-(2-dimethylaminoethyl)-2-phenylindene. The expenses of this work were defrayed from a block grant by the British Empire Cancer Campaign.

### REFERENCES

MORGAN, F. F., MORTON, H. J. AND PARKER, R. C.—(1950) *Proc. Soc. exp. Biol., N.Y.*, **73**, 1.

MOSCONA, A.—(1952) *Exp. Cell Res.*, **3**, 535.

POWELL, A. K.—(1963) *Brit. J. Cancer*, **17**, 293.

WILLMER, E. N.—(1954). 'Tissue Culture'. London (Methuen: Biological Monographs), p. 129.